# Association between Family Histories of Thyroid Cancer and Thyroid Cancer Incidence: A Cross-Sectional Study Using the Korean Genome and Epidemiology Study Data

**DOI:** 10.3390/genes11091039

**Published:** 2020-09-03

**Authors:** Soo-Hwan Byun, Chanyang Min, Hyo-Geun Choi, Seok-Jin Hong

**Affiliations:** 1 Department of Oral & Maxillofacial Surgery, Dentistry, Hallym University College of Medicine, Anyang 14068, Korea; purheit@daum.net; 2 Research Center of Clinical Dentistry, Hallym University Clinical Dentistry Graduate School, Chuncheon 24252, Korea; 3 Hallym Data Science Laboratory, Hallym University College of Medicine, Anyang 14068, Korea; joicemin@naver.com; 4 Department of Otorhinolaryngology-Head & Neck Surgery, Hallym University College of Medicine, Anyang 14068, Korea; 5 Department of Otorhinolaryngology-Head & Neck Surgery, Hallym University College of Medicine, Dongtan 18450, Korea

**Keywords:** epidemiology, thyroid cancer, family history, differentiated thyroid cancer, papillary thyroid cancer

## Abstract

This study assessed the association between thyroid cancer and family history. This cross-sectional study used epidemiological data from the Korean Genome and Epidemiology Study from 2001 to 2013. Among 211,708 participants, 988 were in the thyroid cancer group and 199,588 were in the control group. Trained interviewers questioned the participants to obtain their thyroid cancer history and age at onset. The participants were examined according to their age, sex, monthly household income, obesity, smoking, alcohol consumption, and past medical history. The adjusted odds ratios (95% confidence intervals) for the family histories of fathers, mothers, and siblings were 6.59 (2.05–21.21), 4.76 (2.59–8.74), and 9.53 (6.92–13.11), respectively, and were significant. The results for the subgroup analyses according to sex were consistent. The rate of family histories of thyroid cancer for fathers and siblings were not different according to the thyroid cancer onset, while that of mothers were higher in participants with a younger age at onset (<50 years old group, 11/523 [2.1%], *p* = 0.007). This study demonstrated that thyroid cancer incidence was associated with thyroid cancer family history. This supports regular examination of individuals with a family history of thyroid cancer to prevent disease progression and ensure early management.

## 1. Introduction

The incidence of thyroid cancer is increasing worldwide [1], and it has increased by approximately two folds over the past few decades [2]. The incidence of thyroid nodules diagnosed by palpation of the thyroid gland is approximately 5–10% in adults [3]. Because of the frequent use and accessibility of thyroid ultrasonography (US), there has been an increase in thyroid cancer detection [4]. The objective of the examination of patients with thyroid nodules is to identify thyroid cancer. As a result, its incidence in South Korea in 2011 was 15 times higher than in 1993 [5]. Korean women had the highest age-standardized incidence of thyroid cancer globally [6]. Differentiated thyroid carcinoma (DTC) comprises approximately 90% of all thyroid cancers and includes the papillary type and follicular type [7,8]. In Korea, the most common histological type (94.9%) of DTC is papillary thyroid cancer (PTC) [9]. Total thyroidectomy is usually recommended for treatment of thyroid cancer, followed by radioiodine therapy in some cases. Inoperable or radiotherapy refractory differentiated thyroid cancers are commonly the main causes of thyroid cancer-related deaths and do not have effective treatments [10]. Cytotoxic chemotherapy with innovative medicines should be studied because they appear to be effective in some patients [11,12].

The etiology of DTC is still unknown. However, environmental and genetic predisposing factors including ionizing radiation might affect the development of thyroid cancer [13]. Air pollution and iodine intake are considered risk factors for thyroid cancer [14,15,16,17]. Obesity, smoking, and alcohol consumption are also associated with thyroid cancer [18]. A family history of thyroid cancer is also a suggested risk factor in 5–15% of cases. There are several genetic mutations identified to have a role in the development of DTC [7]. Rearranged during transfection (RET) chromosomal rearrangement genes, and mutation of RAS or BRAF proto-oncogenes can trigger the activation of the mitogen-activated protein kinase cascade in PTC. Mutations of the BRAF, RAS, or RET genes are found in nearly 70% of PTC cases [19,20]. A previous study mentioned that BRAF mutation is associated with aggressiveness of PTC and the loss of radiotherapy effectiveness in recurrent disease. Surgical resection of mutation-positive cancer is recommended [10]. For instance, lobectomy is recommended by the American Thyroid Association for treatment of PTCs < 1 cm [21]. However, because of the definite association of BRAF mutation with aggressiveness of PTC, total thyroidectomy might be a better treatment option if BRAF mutation is positive in preoperative testing [21].

The familial risk of thyroid cancer is known to be highest in all cancer sites, for which the increased risk extends beyond the nuclear family [22,23,24]. Family history is a possible risk factor which could be associated with medical and psychosocial benefits. To provide definite information, the clinicians and entire medical system need to recognize the familial risks that are not included in the conventional familial risk guidelines.

Nevertheless, only few studies have studied the risk factors of familial history for thyroid cancer in Asian adults. Most of the studies were focused on familial non-medullary thyroid cancer, or the study sample sizes were relatively small [25]. Myung et al. reported that a family history of cancer and alcohol consumption were associated with a decreased risk of thyroid cancer, whereas a higher body mass index (BMI) and family history of thyroid cancer were associated with an increased risk of thyroid cancer in 34,211 patients [26]. The study was a case-control study. A total of 802 thyroid cancer cases out of 34,211 patients was included. A total of 802 control cases was also selected from the same cohort group and matched using the ratio 1:1 by age and area of residence. Multivariate conditional logistic regression analysis was used. The results show that females and those with a family history of thyroid cancer had an increased risk of thyroid cancer, and a family history of cancer and alcohol consumption were associated with a decreased risk of thyroid cancer, whereas a higher BMI and family history of thyroid cancer were associated with an increased risk of thyroid cancer. The study suggested that females and those with a family history of thyroid cancer have an increased risk of thyroid cancer. Hwang et al. showed that multicollinearity existed between US assessment and patient age, and first-degree family history of thyroid cancer and serum thyroid hormone values in 1254 patients [25]. A retrospective study investigated 1310 thyroid nodules of 1254 euthyroid asymptomatic patients who underwent US-guided fine needle aspiration. The study evaluated nodule size, first-degree family history of thyroid cancer, gender, age, and thyroid-stimulating hormone (TSH) levels with US examination to distinguish between benign and malignant nodules. Multiple logistic regression analysis was conducted to evaluate the risk of thyroid malignancy according to clinical and US features. A first-degree family history of thyroid cancer, age, and high TSH levels did not independently significantly increase the risk of thyroid cancer. The study concluded that a first-degree family history as a risk factor for thyroid malignancy should be further investigated in asymptomatic patients.

Similar to those studies, previous studies of familial risk were performed without a control group or with a small control group. Moreover, those studies did not include many confounding factors that could influence the study results. To the best of our knowledge, there has been no study on the association between family history and thyroid cancer using large Korean population data.

A clear picture of the association between thyroid cancer and family history will help both patients and clinicians. This could serve as a basis for guidelines governing clinical risk factor assessment and the management of thyroid nodules. Therefore, this study assessed the association between thyroid cancer and family history.

## 2. Materials and Methods

### 2.1. Study Population and Data Collection

Authorization was obtained from the ethics committee of the Hallym University (2019-02-020). The requirement for written informed consent was waived by the Institutional Review Board. This cross-sectional study relied on the data from the Korean Genome and Epidemiology Study (KoGES) from 2001 to 2013.

The Korean Genome and Epidemiology Study Health Examinee (KoGES HEXA) is a large cohort project initiated to reveal gene-environmental factors and their interactions in diseases [27,28]. Among this, we have selected the questionnaires. Therefore, this study had a cross-sectional design. KoGES was designed to identify gene-environment factors and their interactions in common chronic diseases, such as hypertension, cardiovascular disease, type 2 diabetes, obesity, and metabolic syndrome, in Korea. KoGES collected epidemiological data and biospecimens, such as urine, blood, and genome, from many patients aged 40–69 years by performing a medical examination and health survey. Familial data were well-organized and systemized, and the data were collected using a questionnaire, which was confirmed by expert clinicians. The KoGES data were collected from both urban and rural areas. Therefore, the study sample size was relatively larger than that reported in previous studies.

### 2.2. Participant Selection

**Figure 1:**
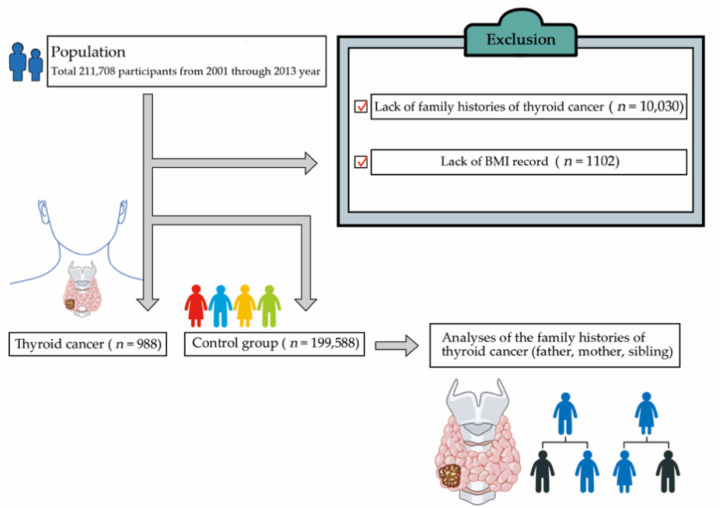
Participant selection.

Among 211,708 participants, we excluded the participants with no family history of thyroid cancer (*n* = 10,030) and no BMI data (*n* = 1102) (Figure 1). In total, 200,576 participants (69,693 men, 130,883 women) were evaluated. According to their cancer thyroid histories, they were divided into two groups: the thyroid cancer group and control group.

### 2.3. Survey

Trained interviewers asked the participants questions to obtain relevant data including previous thyroid cancer history, age at onset, household monthly income, past metabolic disease history (hypertension, diabetes mellitus, and hyperlipidemia), smoking history, and alcohol consumption history. Anthropometric and clinical measurements were obtained [27,28]. This study categorized the family histories of thyroid cancer into groups: fathers, mothers, and siblings (brothers or sisters). Income was categorized into four groups according to the household monthly income: no information, low (<$1500), middle (≥$1500–<$3000), and high (≥$3000). BMI was used to measure obesity (kg/m^2^), wherein height and weight were considered as continuous variables [27,29]. Total smoking histories were calculated in pack-year, and alcohol consumption was measured as the mean daily alcohol consumption (g/day) using the frequency and alcohol types [30,31].

### 2.4. Statistical Analyses

The chi-square test or Fisher’s exact test was used to compare the differences between sex, income, metabolic disease history, and thyroid cancer family history between both groups. The independent *t*-test was used to compare the age, BMI, smoking pack year, and alcohol consumption.

To analyze the odds ratio (OR) of thyroid cancer history for the thyroid cancer group, a logistic regression model was used. In the crude model, this study only inserted each family history of thyroid cancer as an independent variable. In Model 1, this study inserted each family history of thyroid cancer and age, sex, income, BMI, smoking, alcohol intake, and past medical histories of hypertension, diabetes mellitus, and dyslipidemia as independent variables. In Model 2, this study inserted thyroid cancer history of the father, mother, and siblings as independent variables. In Model 3, this study inserted the variables of Models 2 and 3. To analyze the interaction between the different family histories of thyroid cancer, we designed Model 4. In this model, the variables included: thyroid cancer history of father, mother, siblings, father × siblings, and mother × siblings.

The 95% confidence intervals (CIs) were calculated. For the subgroup analysis, this study stratified the participants according to sex: male and female.

Two-tailed analyses were conducted, and *p* values < 0.05 were considered as statistically significant. The results were analyzed using SPSS v. 22.0 (IBM, Armonk, NY, USA).

## 3. Results

The participant age ranged from 40 to 91 years. In total, 988 participants were in the thyroid cancer group, while 199,588 were in the control group. The differences between the mean age, BMI, smoking duration, and daily alcohol consumption of both groups were significant (Table 1). The rates of sex, income, past medical history of diabetes mellitus, and dyslipidemia were also different between both groups.

In Model 1, adjusted for general characteristics, the adjusted ORs for the family histories of fathers, mothers, and siblings were 6.72 (95% CI = 2.10–21.50), 6.33 (95% CI = 3.52–11.36), and 10.16 (95% CI = 7.42–13.93), respectively.

In Model 2, adjusted for only family histories of thyroid cancer, the adjusted ORs for family histories of fathers, mothers, and siblings were 6.75 (95% CI = 2.10–21.67), 5.55 (95% CI = 3.02–10.19), and 13.09 (95% CI = 9.53–17.99), respectively.

In Model 3, adjusted for both general characteristics and family histories of thyroid cancer, the adjusted OR was slightly lower than in Models 1 and 2. The adjusted ORs for the family histories of fathers, mothers, and siblings were 6.59 (95% CI = 2.05–21.21), 4.76 (95% CI = 2.59–8.74), and 9.53 (95% CI = 6.92–13.11), respectively, and were significant (Table 2).

In the subgroup analyses according to sex, the results were consistent with the results of the total participants (Table 3). In men, the adjusted ORs for the family histories of fathers and siblings were 29.09 (95% CI = 3.84–220.29) and 14.15 (95% CI = 3.39–58.95), respectively, in Model 3, while that of mother did not converge. In women, the adjusted ORs for the family histories of fathers, mothers, and siblings were 4.71 (95% CI = 1.13–19.52), 5.06 (95% CI = 2.75–9.31) and 9.37 (95% CI = 6.75–13.00), respectively.

Because no father had a thyroid cancer history when mother had thyroid cancer, we did not make the interaction model for father × mother. In the interaction model, we did not find any interaction in father × siblings and mother × siblings (Table 4). 

The rate of family histories of thyroid cancer of father and siblings were not different according to thyroid cancer onset (Table 5), while that of mother was higher in patients with a younger age at onset (<50 years group, 11/523 [2.1%]) compared with those with an older age at onset (≥50 years group, 1/456 [0.2%], *p* = 0.007).

## 4. Discussion

The definite association between family history and thyroid cancer incidence is not yet completely known. Although most cases of PTC occur sporadically, it seems that there are family components in some cases of PTC. Familial non-medullary thyroid cancer (FNMTC) is defined as a condition in which two or more first-degree relatives are affected by thyroid cancer in the absence of a known familial syndrome [32,33]. FNMTC shows a tendency to be more aggressive than sporadic cases with higher rates of extra-thyroid extension, lymph node metastases, larger tumor size in younger patients, and worse prognosis [32,33]. This study focused on the family history of PTC in a large population and not FNMTC. The most common histological type (94.9%) of DTC in Korea was PTC [9]. Most participants with thyroid cancer in the KoGES data would have PTC based on the results of previous studies [9,34,35,36]. There are few studies on the family history of PTC in a large population. Therefore, this study investigated the association between thyroid cancer and family history using data from a large Korean study.

This study showed that the adjusted OR for family history was higher in all thyroid cancer patients than in the control group (Table 2). Family history was significantly associated with the incidence of thyroid cancer after adjustment for age, sex, income, hypertension, diabetes, dyslipidemia, obesity, smoking, and alcohol consumption (Table 1). A meta-analysis of seven cohort studies by Zhao et al. showed that obesity increased the risk of thyroid cancer [37]. The meta-analysis evaluated the association between body weight or BMI and risk of thyroid cancer. A total of 5154 thyroid cancer cases was included. The pooled relative risk (RR) of thyroid cancer was 1.13 (95% CI 1.04–1.22) for overweight. Obesity was related with increased thyroid cancer risk in both genders, the strength of the association increasing with increasing BMI. The combined RR of thyroid cancer was 1.18 (95% CI 1.11–1.25) for excess body weight. Being overweight was associated with a significant increase in the thyroid cancer risk among non-Asians, but not among Asians. Overweight, obesity, and excess body weight were linked to PTC risk. Han et al. reported that obesity was associated with a higher prevalence of thyroid cancer in women [38]. The study collected data from 15,068 subjects who received a health examination from 2007 to 2008 at the Health Screening and Promotion Center of Asan Medical Center in Korea. Thyroid US was conducted in the examination, and suspected nodules were additionally examined by US-guided aspiration. Those with a history of thyroid disease or family history of thyroid cancer were excluded from the study. In total, 15,068 participants were screened by thyroid US. The prevalence of thyroid cancer in females was related with a high BMI (per 5 kg/m^2^ increase) (OR = 1.63, 95% CI 1.24–2.10, *p* < 0.001) after adjustment for age, smoking status, and TSH levels. There was no significant correlation between the prevalence of thyroid cancer in males and a high BMI (OR = 1.16, 95% CI 0.85–1.57, *p* = 0.336). There was no association between age, fasting serum insulin, or basal TSH levels and thyroid cancer in either gender. In a meta-analysis of observational studies, Cho et al. showed that the risk of thyroid cancer was decreased by 21% in smokers compared to non-smokers [39]. The study investigated 31 studies to analyze the relationship between thyroid cancer occurrence and smoking. These studies consisted of 6260 thyroid cancer cases and 32,935 controls. The cohort studies included 2715 thyroid cancer patients. Summary RRs and 95% CIs were calculated using a random effects model. The risk of thyroid cancer was decreased in participants with a past smoking history (RR = 0.79; 95% CI 0.70–0.88) compared with those without. However, strong evidence of heterogeneity was found among the investigated studies; therefore, subgroup analyses were performed according to study location, study type, source of controls, smoking status, sex, and histological type of thyroid cancer. When the data were stratified by smoking status, an inverse association was observed only among current smokers (RR = 0.74; 95% CI 0.64–0.86), not former smokers (RR = 1.01; 95% CI 0.92–1.10). Previous studies demonstrated that alcohol consumption was found to be significantly associated with a decreased risk of thyroid cancer when the analysis was performed using the control group [40,41,42]. Kitahara et al. evaluated data from five prospective United States studies (384,433 males and 361,664 females) [40]. Hazard ratios and 95% CIs for thyroid cancer were calculated from adjusted models of smoking and alcohol consumption with additional adjustment of age, sex, race, education, and BMI. In total, 1003 thyroid cancer cases (335 males and 668 females) were identified. Alcohol intake was also inversely associated with thyroid cancer risk (≥7 drinks/week versus 0, HR = 0.72, 95% CI 0.58–0.90). Inverse associations with alcohol consumption were more pronounced for papillary versus follicular tumors. Hong et al. investigated 33 observational studies with two cross-sectional studies, 20 case-control studies, and 11 cohort studies [42]. The studies involved 7725 thyroid cancer participants and 3,113,679 participants without thyroid cancer. In the fixed-effect model meta-analysis of all 33 studies, alcohol intake was related with a reduced risk of thyroid cancer (OR = 0.74; 95% CI 0.67–0.83). In the subgroup meta-analysis, alcohol consumption also reduced the risk of thyroid cancer in both case-control studies (OR = 0.77; 95% CI 0.65–0.92) and cohort studies (RR = 0.70; 95% CI 0.60–0.82). Subgroup meta-analyses showed that alcohol consumption was significantly related with a reduced risk of thyroid cancer.

The present study adjusted for various confounding factors to reduce the surveillance bias. The adjusted ORs in this study were similar to those in previous studies performed in other countries [7,8,23]. Kust et al. reported that family history plays a significant role in the development of thyroid cancer. Having first-degree relatives with thyroid cancer is a risk factor in both medullary and papillary thyroid cancer. The first-degree relatives could predict the risk of thyroid cancer [7]. A total of 10,709 participants was included in the study. Correlation of cytological findings and family history was evaluated using Fisher’s exact test. There were 2580 (24.09%) patients with non-malignant thyroid diseases in the family and 198 (1.85%) patients with a history of thyroid cancer in the family. A total of 2778 (25.94%) patients had a positive family history of thyroid diseases, and 7931 (74.06%) patients had a negative family history. In patients with a family history of papillary thyroid carcinoma, the difference between those with benign and malignant thyroid tumors was found to be significant (*p* = 0.0432). Thyroid cancer may be more aggressive in younger patients and may have a higher rate of lymph node metastasis [7]. The rates for family histories of fathers and siblings were not different according to the age at onset of thyroid cancer, while those of mothers were higher in patients with a younger age at onset of thyroid cancer (Table 5). In addition, this study analyzed the interaction among familial histories by using the interaction model. There was no significant interactional effect in father with siblings and mother with siblings (Table 4).

Despite our large sample size, this study has few limitations. First, it was impossible to consider all the confounding factors for the association. KoGES did not cover all of the potentially influencing factors such as history of radiation therapy and CT, iodine intake, and history of thyroiditis. Second, KoGES HEXA was started based on a questionnaire survey [27]. The patient’s thyroid cancer history was asked. However, in the cases of positivity, no biopsy or ultrasonography was performed for a definitive diagnosis. This study included all participants who had thyroid cancer as PTC given that about 95% of DTC cases in Korea were PTC [9]. Third, this study included only the participants who were survivors after being diagnosed with thyroid cancer. The survey could not be performed with the dead participants. However, this was not a huge problem because the survival rate of thyroid cancer in Korea is high. Thyroid cancer has an excellent prognosis and a five-year relative survival rate of 100.1% in Korea [43]. Lastly, this study did not include the family history of grandparents or distant relatives. Last, the reliability of the questionnaires on the smoking, alcohol consumption, and nutritional intake frequencies were unclear [27]. To collect accurate data, the reliability and validity of the questionnaire survey should be examined in future studies.

In contrast, this study has several advantages. To the best of our knowledge, this study is the first population-based study examining the association between thyroid cancer family history and the incidence of thyroid cancer in Asia. Second, this study is a large population-based study compared with studies in other countries. This study investigated detailed associations in subgroups in a large population. In addition, the risk of family history on incidence was evaluated using a large control group. This study provides more precise information than most previous individual studies. Third, this study considered many more influential factors than in previous studies. Obesity, smoking, and alcohol consumption were further adjusted in this study. These confounding factors would be important adjustments for the analysis of family history as a risk factor.

## 5. Conclusions

This study demonstrated that the incidence of thyroid cancer was associated with thyroid cancer family history. This finding supports regular examination of individuals with a family history of thyroid cancer to prevent the progression of thyroid cancer. The identification of family history would provide opportunities for early detection and prevention. Further studies including those on gene mutations associated with family history are recommended to demonstrate the pathophysiology and prevalence of thyroid cancer.

## Figures and Tables

**Table 1:** General characteristics of participants.

Characteristics	Total Participants		
	Thyroid Cancer (*n*, %)	Control (*n*, %)	*p*-Value
Total number (*n*, %)	988 (100.0)	199,588 (100.0)	
Age (years, mean, SD)	53.2 (7.6)	53.9 (8.7)	0.007 ^1^
Sex (*n*, %)			<0.001 ^1^
Male	79 (8.0)	69,614 (34.9)	
Female	909 (92.0)	129,974 (65.1)	
Income (*n*, %)			<0.001 ^1^
No information	95 (9.6)	40,590 (20.3)	
Low	166 (16.8)	41,060 (20.6)	
Middle	292 (29.6)	51,899 (26.0)	
High	435 (44.0)	66,039 (33.1)	
Hypertension (*n*, %)	220 (22.3)	40,930 (20.5)	0.172
Diabetes (*n*, %)	50 (5.1)	14,584 (7.3)	0.007 ^1^
Dyslipidemia (*n*, %)	117 (11.8)	17,834 (8.9)	0.001 ^1^
Obesity (BMI, kg/m^2^, mean, SD)	23.8 (2.9)	24.0 (3.0)	<0.001 ^1^
Tobacco index (pack-year, mean, SD)	1.63 (6.5)	6.62 (12.5)	<0.001 ^1^
alcohol consumption (g/day, mean, SD)	2.0 (10.1)	7.2 (22.1)	<0.001 ^1^
Family history of father (*n*, %)	3 (0.3)	86 (0.0)	0.010 ^1^
Family history of mother (*n*, %)	12 (1.2)	307 (0.2)	<0.001 ^1^
Family history of siblings (*n*, %)	44 (4.5)	653 (0.3)	<0.001 ^1^

BMI, body mass index, kg/m^2^; SD, Standard deviation. ^1^ Independent *t*-test, Chi-square test, or Fisher’s exact test. Significance at *p* < 0.05.

**Table 2:** Association between thyroid cancer history and their family thyroid cancer histories.

Family History	ORs of Thyroid Cancer for the Thyroid Cancer Family Histories							
	Crude	*p*-Value	Model 1	*p*-Value	Model 2	*p*-Value	Model 3	*p*-Value
ORs of the thyroid cancer history of father								
Thyroid cancer	7.07 (2.23–22.38)	0.001 ^1^	6.72 (2.10–21.50)	0.001 ^1^	6.75 (2.10–21.67)	0.001 ^1^	6.59 (2.05–21.21)	0.002 ^1^
Control	1.00		1.00		1.00		1.00	
ORs of the thyroid cancer history of mother								
Thyroid cancer	7.98 (4.47–14.26)	<0.001 ^1^	6.33 (3.52–11.36)	<0.001 ^1^	5.55 (3.02–10.19)	<0.001 ^1^	4.76 (2.59–8.74)	<0.001 ^1^
Control	1.00		1.00		1.00		1.00	
ORs of the thyroid cancer history of siblings								
Thyroid cancer	14.20 (10.40–19.40)	<0.001 ^1^	10.16 (7.42–13.93)	<0.001 ^1^	13.09 (9.53–17.99)	<0.001 ^1^	9.53 (6.92–13.11)	<0.001 ^1^
Control	1.00		1.00		1.00		1.00	

ORs, odds ratios. Model 1: adjusted for age, sex, income, body mass index, smoking, alcohol intake, and past medical histories of hypertension, diabetes mellitus, and dyslipidemia. Model 2: adjusted for thyroid cancer history of father, mother, and siblings. Model 3: adjusted for Models 1 and 2. ^1^ Logistic regression analyses, Statistical significance at *p* < 0.05.

**Table 3:** Subgroup analyses of association between thyroid cancer history and their family thyroid cancer histories according to sex.

Family History	ORs of Thyroid Cancer for the Thyroid Cancer Histories of Families							
	Crude	*p*-Value	Model 1	*p*-Value	Model 2	*p*-Value	Model 3	*p*-Value
**Men (*n* = 69,693)**								
ORs of the thyroid cancer history of father								
Thyroid cancer	27.88 (3.76–206.55)	0.001 ^1^	28.21 (3.73–213.69)	0.001 ^1^	28.53 (3.85–211.44)	0.001 ^1^	29.09 (3.84–220.29)	0.001 ^1^
Control	1.00		1.00		1.00		1.00	
ORs of the thyroid cancer history of mother								
Thyroid cancer	No convergence	0.997	No convergence	0.997	No convergence	0.997	No convergence	0.997
Control	1.00		1.00		1.00		1.00	
ORs of the thyroid cancer history of siblings								
Thyroid cancer	15.43 (3.75–63.54)	<0.001 ^1^	13.90 (3.34–57.94)	<0.001 ^1^	16.02 (3.89–66.00)	<0.001 ^1^	14.15 (3.39–58.95)	<0.001 ^1^
Control	1.00		1.00		1.00		1.00	
**Women (*n* = 130,883)**								
ORs of the thyroid cancer history of father								
Thyroid cancer	5.31 (1.29–21.79)	0.021 ^1^	4.84 (1.18–19.94)	0.029 ^1^	4.92 (1.18–20.61)	0.029 ^1^	4.71 (1.13–19.52)	0.033 ^1^
Control	1.00		1.00		1.00		1.00	
ORs of the thyroid cancer history of mother								
Thyroid cancer	7.89 (4.40–14.16)	<0.001 ^1^	6.75 (3.75–12.14)	<0.001 ^1^	5.70 (3.09–10.51)	<0.001 ^1^	5.06 (2.75–9.31)	<0.001 ^1^
Control	1.00		1.00		1.00		1.00	
ORs of the thyroid cancer history of siblings								
Thyroid cancer	11.70 (8.49–16.13)	<0.001 ^1^	10.04 (7.27–13.87)	<0.001 ^1^	10.81 (7.80–14.98)	<0.001 ^1^	9.37 (6.75–13.00)	<0.001 ^1^
Control	1.00		1.00		1.00		1.00	

Model 1: adjusted for age, sex, income, body mass index, smoking, alcohol intake, and past medical histories of hypertension, diabetes mellitus, and dyslipidemia. Model 2: adjusted for thyroid cancer history of father, thyroid cancer history of mother, and thyroid cancer history of siblings. Model 3: adjusted for Models 1 and 2. ^1^ Logistic regression analyses, Statistical significance at *p* < 0.05.

**Table 4:** Interaction model of each family history of thyroid cancer.

Variable	ORs for Thyroid Cancer	
	Model 4	*p*-Value
Father	No convergence	1.00
Mother	12.84 (2.02–81.86)	0.007
Siblings	26.87 (5.69–126.91)	<0.001
Father × siblings	No convergence	1.000
Mother × siblings	0.51 (0.12–2.17)	0.362

Model 4: adjusted for thyroid cancer history of father, mother, siblings, father × siblings, and mother × siblings.

**Table 5:** Ratio of family histories of thyroid cancer according to thyroid cancer onset among the thyroid cancer participants.

Histories	Onset of Thyroid Cancer		
	<50 Years Old	≥50 Years Old	*p*-Value
Thyroid cancer histories of father (*n*, %)			
Yes	3 (0.6)	0 (0.0)	0.253
No	520 (99.4)	456 (100.0)	
Thyroid cancer histories of mother (*n*, %)			
Yes	11 (2.1)	1 (0.2)	0.007 ^1^
No	512 (97.9)	455 (99.8)	
Thyroid cancer histories of siblings (*n*, %)			
Yes	23 (4.4)	21 (4.6)	0.876
No	500 (95.6)	435 (95.4)	

^1^ Fisher’s exact test. Significance at *p* < 0.05.
